# Multi-probe attention neural network for COVID-19 semantic indexing

**DOI:** 10.1186/s12859-022-04803-x

**Published:** 2022-06-29

**Authors:** Jinghang Gu, Rong Xiang, Xing Wang, Jing Li, Wenjie Li, Longhua Qian, Guodong Zhou, Chu-Ren Huang

**Affiliations:** 1 Department of Chinese and Bilingual Studies, The Hong Kong Polytechnic University, Hong Kong, China; 2 Department of Computing, The Hong Kong Polytechnic University, Hong Kong, China; 3 Tencent AI Lab, Shenzhen, China; 4 School of Computer Science and Technology, Soochow University, Suzhou, China

**Keywords:** COVID-19, Topic identification, Biomedical semantic indexing, Deep learning

## Abstract

**Background:**

The COVID-19 pandemic has increasingly accelerated the publication pace of scientific literature. How to efficiently curate and index this large amount of biomedical literature under the current crisis is of great importance. Previous literature indexing is mainly performed by human experts using Medical Subject Headings (MeSH), which is labor-intensive and time-consuming. Therefore, to alleviate the expensive time consumption and monetary cost, there is an urgent need for automatic semantic indexing technologies for the emerging COVID-19 domain.

**Results:**

In this research, to investigate the semantic indexing problem for COVID-19, we first construct the new COVID-19 Semantic Indexing dataset, which consists of more than 80 thousand biomedical articles. We then propose a novel semantic indexing framework based on the multi-probe attention neural network (MPANN) to address the COVID-19 semantic indexing problem. Specifically, we employ a k-nearest neighbour based MeSH masking approach to generate candidate topic terms for each input article. We encode and feed the selected candidate terms as well as other contextual information as probes into the downstream attention-based neural network. Each semantic probe carries specific aspects of biomedical knowledge and provides informatively discriminative features for the input article. After extracting the semantic features at both term-level and document-level through the attention-based neural network, MPANN adopts a linear multi-view classifier to conduct the final topic prediction for COVID-19 semantic indexing.

**Conclusion:**

The experimental results suggest that MPANN promises to represent the semantic features of biomedical texts and is effective in predicting semantic topics for COVID-19 related biomedical articles.

## Introduction

With COVID-19 sweeping across the world, the challenge of the pandemic has rapidly accelerated the pace of scientific publications [1, 2]. As approximately 10,000 new articles on COVID-19 and SARS-CoV-2 are published every month [3], the ability to accurately extract the crucial semantic topics from the large rapidly-growing COVID-19 literature has become of great importance to many biomedical applications [4–7].

**Fig. 1:**
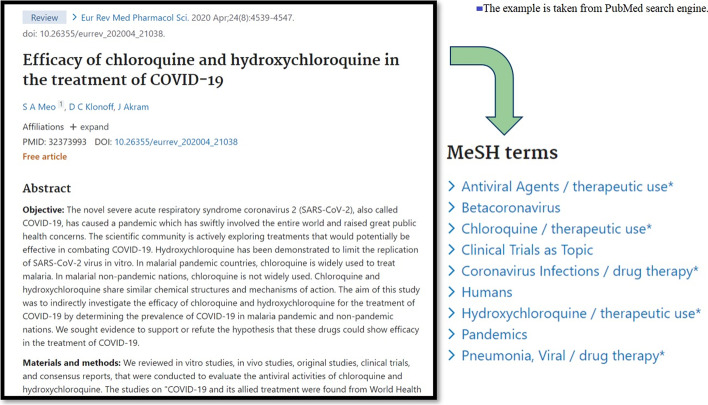
An example of MeSH semantic indexing taken from PubMed

In recent decades, curators at the National Library of Medicine (NLM) have been employing Medical Subject Headings (MeSH) to manually identify and curate semantic topics for scientific articles [8–10], which is also known as the process of semantic indexing. However, it is non-trivial to manually curate such substantial biomedical articles, which heavily relies on intensive labour and tremendous investment. In this scenario, experts have to examine the full body of each biomedical article and manually assign it with a series of suitable pre-defined semantic topic terms from the large vocabulary of MeSH headings. Although this manual topic assignment has relatively reliable accuracy, it is inevitably time-consuming and prohibitively expensive [11–13]. In addition, due to the emerging hotspots of COVID-19, such manual topic curation is much more difficult to keep up to date. Moreover, lacking a pertinent biomedical taxonomy will further increase the challenges of the topic curation for COVID-19. Hence, there is an urgent need for automatic semantic indexing techniques that are able to efficiently and robustly identify biomedical topics in a newly emerged topical field, such as the COVID-19 domain. Figure 1 shows an example to illustrate the challenges of the semantic indexing task for the COVID-19 domain. In the figure, the article (PMID: 32,373,993) has already been curated and indexed by MEDLINE experts with nine different MeSH semantic topics.

In view of machine learning, automatic semantic topic indexing with MeSH terminologies is considered a large-scale multi-label topic identification problem. Despite the promising results from early efforts [14–17], there is still a significant gap between such automatic methods and their applications for effective searching and querying in the COVID-19 domain. On the one hand, there is a lack of a specialized biomedical taxonomy for COVID-19 as traditional MeSH indexing research concentrates on the general scientific domains. Even worse, with tens of thousands of topic terms in the large-scale vocabulary of MeSH headings, it almost inevitably leads to extremely imbalanced label distribution for the ground-truth semantic topics [17]. On the other hand, there is also a severe lack of benchmark datasets for the COVID-19 semantic indexing research. At present, fighting the COVID-19 pandemic poses an extreme scenario that highlights the importance of automated semantic indexing techniques as professionals and practitioners desperately require a well-structured knowledge base to acquire new insights from recent coronavirus findings [18–20]. However, lacking such a standard dataset drastically limits the development of the topic identification techniques for the COVID-19 domain. Therefore, constructing a universal dataset for COVID-19 semantic indexing is of great importance.

In light of these concerns, this article is devoted to the topic identification problem of COVID-19 semantic indexing. Theoretically, the COVID-19 semantic indexing can be conceptualized as a typical case of labeling texts with a range of centralized topics from heterogeneous sources. The need for such kind of semantic labeling is crucial for an emerging thematic area. Typically, neither consensus domain taxonomy nor sufficient annotated training data are available in such emerging topical areas. In addition, such an emerging domain also lacks a conventionalized venue for publications and likely finds a variety of related publications in neighboring fields. In this regard, we first introduce a new COVID-19 Semantic Indexing (CovSI) corpus constructed from a wide range of COVID-19 related biomedical articles, which addresses the data absence in such an emerging domain. We then propose a novel deep neural network adopting a multi-probe attention mechanism to address the challenges of semantic indexing from heterogeneous data for the specific field, i.e., COVID-19. Since there is no such specialized topic taxonomy for COVID-19 so far, the classic and widely used MeSH controlled vocabulary is employed for the study. To construct the CovSI corpus, we extract the metadata from multiple authoritative resources, including MEDLINE [12], PubMed Central (PMC) [21], and COVID-19 Open Research Dataset (CORD-19) [1], respectively. All extracted metadata is then merged to build the CovSI corpus. On top of the CovSI corpus, we propose a novel semantic indexing framework based on multi-probe attention neural network (MPANN) to address the fundamental problem of semantic indexing for the emerging domain of COVID-19. The proposed method begins by ranking all MeSH topic terms for each article through a k-nearest neighbor (KNN) based masking approach, which is able to select the most relevant candidate topics and significantly reduce the complexity of the MeSH controlled vocabulary without any prior knowledge of the domain. It then represents multiple context-aware inputs for potential biomedical clues with a transformer encoder and subsequently feeds the encoded representations to the downstream attention-based neural network for further feature extraction. Specifically, four different semantic probes, namely Context Probe, Candidate Term Probe, Journal Probe, and Dynamic Topic Probe, are exploited during the feature extraction phase in order to address the heterogeneous nature of the data sources. The basic idea of these probes relies on that the context-aware textual information carries meaningful biomedical background knowledge from different semantic aspects, which provides informative features to discriminate topics for the input article. For instance, COVID-19 related literature is likely to express the conceptional terminologies of *Coronavirus* and *SARS-CoV-2*, which are suggestive indicators for topic selection. In this view, associating the expressive contexts with the sieved candidate topic terms can help the MPANN model pay more attention to the possible target topics during the classification. Moreover, given a wide variety of sources of publications, COVID-19 articles may allow attention directly to the journals that are most likely within a relationship to the specific topic, such as journals on respiratory diseases for COVID-19. After extracting the feature representations at both term-level and document-level, MPANN adopts a linear multi-view classifier to conduct the final MeSH recommendation. To improve the overall performance, the proposed method is pre-trained using a large number of MEDLINE articles to learn the general biomedical representation, and further fine-tuned on the CovSI dataset to better obtain COVID-19 related knowledge.

Our primary goal is to construct a publicly available dataset for the COVID-19 semantic indexing research and develop a versatile machine learning approach with robustness and generalizability, which can be easily applied to COVID-19 and robustly scaled up to other biomedical domains, especially those new emerging topics. Experimental results on the dataset show the merit and effectiveness of our proposed approach in such a specific domain of COVID-19. The main contributions of this work are summarized as follows:

- We construct a pertinent and comprehensive corpus targeting the COVID-19 semantic indexing research. We believe such a corpus could largely benefit the related works for COVID-19 and foster the development of biomedical text mining technologies.
- We propose a novel semantic indexing approach that is able to effectively scale up to the COVID-19 domain. Our study demonstrates the superiority of the proposed method which outperforms the current state-of-the-art performance.
- We make the related resources of the proposed method publicly available to the research community. We believe that our work is capable of offering some essential foundations for researchers under the current pandemic crisis.

## Related work

In recent decades, to facilitate the research of biomedical topic curation, a series of automated methods [22–32] and challenging competitions [33, 34] have been developed to improve the time-consuming, costly, and labor-intensive semantic indexing process.

Learning-to-rank (LTR) is one of the most popular information retrieval approaches developed for semantic indexing [35]. The main idea of LTR is to model the topic identification problem as a ranking problem, where the top-ranked semantic topics are recommended as true labels. To this end, NLM developed the famous retrieval tool Medical Text Indexing (MTI) [13, 22], which has been assisting NLM human curators since 2002. Specifically, MTI has two separate components: MetaMap Indexing and PubMed Related Citations. Once texts from a biomedical article are fed into MTI, it automatically recommends suitable MeSH topics to the human curators.

To encourage worldwide research on biomedical topic curation, a series of semantic indexing competitions have been held annually by the BioASQ community since 2013 [33]. Participants involved are required to predict new MEDLINE articles with relevant MeSH topics. As the competitions have provided large-scale practical and realistic benchmarks, many efficacious studies have emerged since then. MeSHLabeler [23] developed an LTR-based hybrid system with textual representations for multiple integrated classifiers. To handle the prediction bias generated by the integrated classifiers, MeSHLabeler adopted a normalization schema to improve prediction accuracy and won first place in the BioASQ 2014 competition. MeSHNow [24] proposed another hybrid machine learning approach, which combined multi-label classification, KNN, and MTI, to generate the set of candidate MeSH terms for each article. Under the effectiveness of the LTR-based framework, MeSHNow successfully extracted the highest-ranked semantic topics and reached the state-of-the-art performance on the BioASQ 2014 dataset.

With the success of deep neural networks [36–40], deep learning-based approaches have brought remarkable breakthroughs in various biomedical semantic indexing tasks [25–30]. DeepMeSH [27] proposed a neural semantic representation method to address the BioASQ 2015 semantic indexing task. It first utilized the feature representations of ‘document to vector’ (D2V) and ‘term frequency with inverse document frequency’ (TFIDF) to tackle the topic selection problem. It then ranked the identified topics via an LTR-style framework to determine the final MeSH recommendation. FullMeSH [28] took advantage of an Attention-based Convolution Neural Network (AttentionCNN) to tackle the large-scale semantic indexing problem. Specifically, it combined the AttentionCNN with traditional machine learning methods (including KNN, SVM, etc.) to generate semantic evidence for the topic selection problem. Instead of manual feature engineering, the attention mechanism exhibited remarkable potential on account of an automatic feature representation without too much human interference. Benefiting from the AttentionCNN structure, all evidence extracted from the full text is fused into the downstream LTR module to conduct the final MeSH recommendation. AttentionMeSH [29] was another effective attention-based neural model. It utilized a bidirectional Recurrent Neural Network (RNN) with an attention mechanism to index MeSH topics for biomedical articles. It first narrowed down the large MeSH vocabulary through a masking method and then employed the RNN to derive deeper contextual representations. As a result of the capability of the deep neural representation, AttentionMeSH enabled the model to associate more textual evidence with plausible MeSH topics. MeSHProbeNet [25] and MeSHProbeNet-P [26] are two homogenous deep learning methods, which incorporated both RNN and attention mechanisms. The main difference between the two methods is that MeSHProbeNet-P presented multiple semantic probes as inputs based on MeSHProbeNet, which is able to acquire deeper semantic insights into biomedical knowledge from original plain texts. Contrasting the LTR-based models, MeSHProbeNet and MeSHProbeNet-P take the entire topic vocabulary of MeSH headings to perform the unified multi-label classification without any ranking solutions. Both MeSHProbeNet and MeSHProbeNet-P reached state-of-the-art performance on the dataset of BioASQ 2018 Task8a, and MeSHProbeNet won first place during the online competition.

Recently, in response to the worldwide pandemic, the focus of research has drastically shifted towards the specific concepts and sub-concepts of coronavirus. The BioCreative-VII community proposed the challenging task of the LitCovid Track [34], which targets identifying semantic topics to the COVID-19 relevant literature. Accordingly, the LitCovid task is regarded as a multilabel classification problem and engaged worldwide efforts to provide practical benefits to the COVID-19 topic curation. In particular, seven elaborated semantic topics, i.e., *Treatment*, *Diagnosis*, *Prevention*, *Mechanism*, *Transmission*, *Epidemic Forecasting*, and *Case Report*, are designated for the task. However, although advanced participating systems [31, 32] achieved remarkable performance in the LitCovid challenge, such a small set of coarse-grained semantic topics still limits its applications to real-world scenarios. In contrast, BioTrans [30] suggested leveraging the MeSH taxonomy to enrich the topic abundance for COVID-19 topic curation. Specifically, BioTrans explored a sophisticated pre-trained transformer to address the COVID-19 topic identification problem. With the powerful representation capability of the transformer, BioTrans exhibited a promising achievement in the COVID-19 relevant literature. However, the lack of publicly available benchmark datasets still remains challenging when transferring recent advances to the newly emerged COVID-19 domain, as models cannot be re-trained and fine-tuned without adequate annotations.

Inspired by previous research [26, 30, 34], this article is devoted to the COVID-19 semantic indexing problem. Our goal is to develop a benchmark dataset and a robust yet flexible semantic topic identification framework for the COVID-19 domain, which has not been addressed in previous research.

### Dataset

In this section, a new dataset of the COVID-19 Semantic Indexing (CovSI) corpus is illustrated. Specifically, we first depict its construction steps, and then we present the data statistics accordingly.

### Corpus construction

Since there is a lack of specialized datasets for COVID-19 semantic indexing, it is of great importance to build such a corpus, laying the foundation for research. In this article, we utilize various kinds of existing COVID-19 related resources to construct such a corpus.

As the COVID-19 Open Research Dataset (CORD-19) [1] provides the largest COVID-19 relevant dataset, it is natural to be leveraged as the fundamental resource for the construction of the CovSI corpus due to its expansive coverage and public accessibility. Currently, CORD-19 consists of more than 500,000 scholarly articles related to COVID-19, SARS-CoV-2, and other coronaviruses collected from more than 3,200 journals. However, although CORD-19 carries lots of fundamental ingredients for CovSI (e.g. titles and abstracts), it does not provide any relevant clues for handling semantic indexing problems, which brings difficulties to building such a benchmark dataset.

To complement the indexing annotations for the CovSI corpus, the worldwide used databases curated by the PubMed search engine are considered as the preferred supplementation. Specifically, databases of MEDLINE [12] and PMC [21] indexed by PubMed are employed in this research. MEDLINE is a large bibliographic database that contains more than 27 million scientific references with titles and abstracts, while PMC is a full-text derived biomedical collection that curates more than 6 million publicly available articles. Unlike CORD-19, which is merely concentrated on the topics of coronavirus, MEDLINE and PMC present a more comprehensive subject scope and carry the essential semantic indexing annotations for CovSI.

On the basis of the above-described resources, we propose to extract the metadata from each resource and merge them to construct the new benchmark dataset of the CovSI corpus. However, regarding the heterogeneous data structures among different resources, data inconsistency and incompleteness are therefore crucial to be tackled during the construction phase. For instance, PMIDs/PMCIDs are treated as the unique keys for articles in MEDLINE and PMC, while some are occasionally missing for the articles curated by CORD-19, leading to an inability to map these articles. Moreover, CORD-19 does not provide any information for semantic indexing, while the metadata from MEDLINE and PMC do support the critical annotations for MeSH terms.

**Fig. 2:**
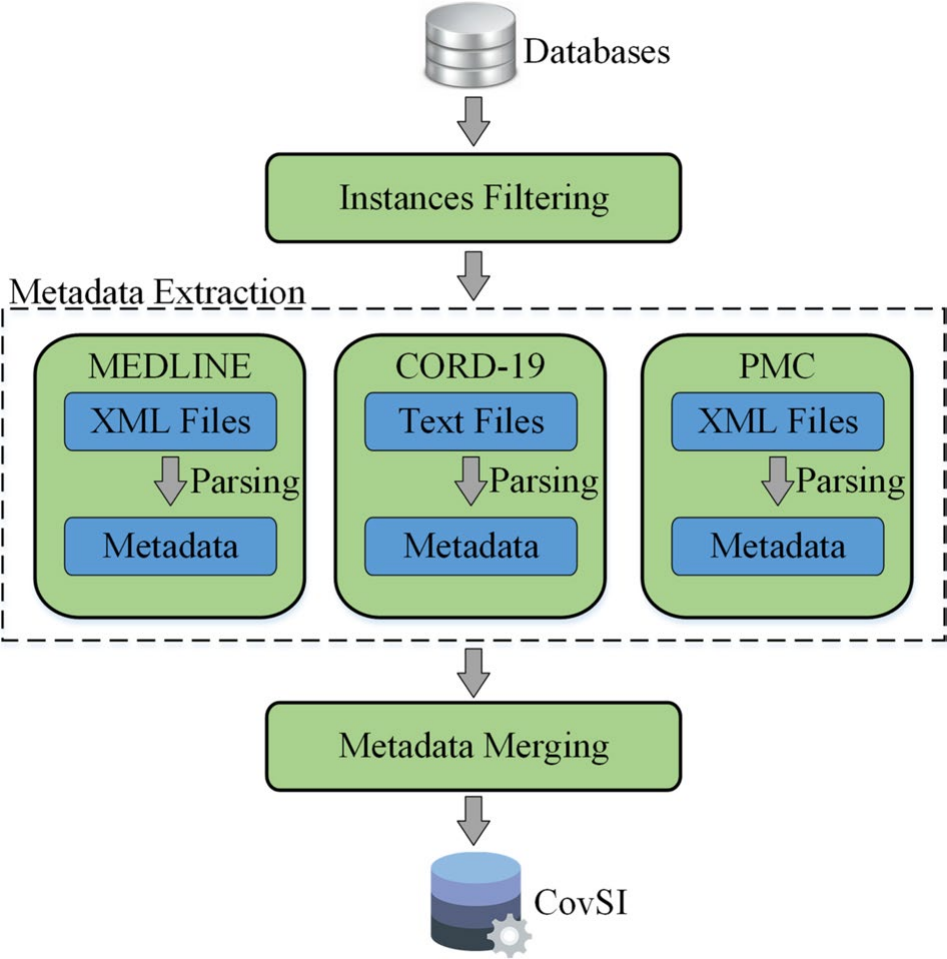
The construction framework of the CovSI corpus

Figure 2 depicts the construction architecture of the CovSI corpus. Note that the keys of PMID and PMCID are used as unique identifiers when extracting and mapping the metadata from different resources. In the figure, we first extract all various kinds of attribute fields from different databases, we then filter the redundant information and reserve the extracted attribute fields as new metadata. During the extraction phase, articles without valid PMIDs or PMCIDs are discarded. After merging the extracted metadata, the CovSI corpus is finally constructed. It is worth noting that all contents in the CovSI corpus are converted and stored in the JSON format, which is one of the most effective and widely used archive formats for data usage and storage.

### Corpus analysis

Table 1 presents the statistical information of the constructed CovSI corpus. After the metadata merging, there are 87,207 COVID-19 related biomedical articles reserved in the CovSI corpus. Each article contains 15 different attribute fields, such as PMID, title, abstract, body text, journal name, and MeSH terms. These abundant attributes assure comprehensive coverage for research on COVID-19 topics. Most of the curated articles are filled with valid contents, including title, abstract, journal name, as well as MeSH annotations, which guarantee the indispensable information for the downstream semantic indexing research. A large number of 1,161,962 MeSH topic terms with more than 10 thousand unique term types are kept as annotations in the corpus. However, despite trying the best to fill the attributes, approximately 50% of body texts, keywords, and chemical information are still missing due to the incompleteness of the online information. It is observed that articles have around 13 indexed MeSH terms on average, which indicates an extremely imbalanced term distribution, as most MeSH terms may never be observed in an article.

**Table 1:** The attribute statistics in the CovSI corpus

Attribute name	Count
PMID	87,207
PMCID	46,487
Title	87,192
Abstract	87,162
Body Text	45,968
MeSH Terms	1,161,962
MeSH Identifiers	1,161,962
Journal Name	87,207
Year	87,207
Authors	87,128
Affiliations	83,749
Keywords	35,928
Chemicals	43,711
DOI	77,776
URL	87,207

After the data construction, the CovSI corpus is further randomly divided into three subsets by the ratio of 8:1:1, which indicates the training set, development set, and test set, respectively. Table 2 shows the statistics of the three subsets. Note that each article is able to bring around 13 MeSH terms on average, which guarantees a similar term distribution for all subsets. The CovSI corpus will be freely available to global research communities for applying recent advances in natural language processing and other artificial intelligence techniques to generate new insights in support of the ongoing fight against the pandemic.

**Table 2:** The statistic information of different CovSI datasets

Type	Training set	Development set	Test set
#Articles	71,207	8,000	8,000
#MeSH term types	17,758	9,035	8,991
#Total terms	945,462	106,088	110,412
#Average terms per article	13.28	13.26	13.80

## Method

**Fig. 3:**
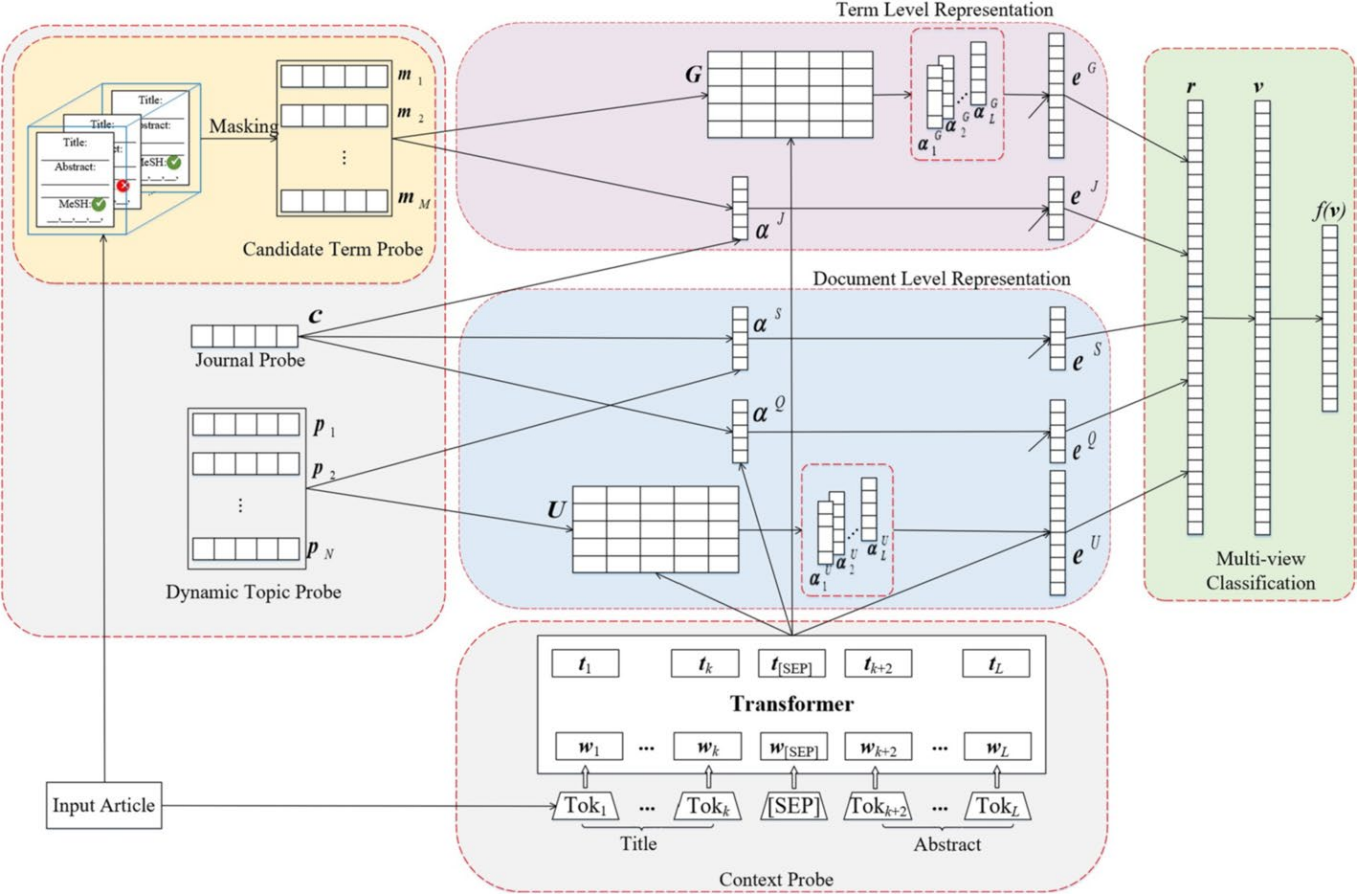
The framework of the multi-probe attention neural network

In this section, a novel Multi-Probe Attention Neural Network (MPANN) is proposed for automatic COVID-19 semantic indexing. Figure 3 illustrates the architecture of the proposed method, which is a universal deep learning framework integrating multiple semantic evidence generated by different biomedical aspects. The architecture of MPANN mainly consists of four modules: *MeSH Masking*, *Probe Encoding*, *Multi-Probe Attention,* and *Multi-view Classifier*. The details are discussed as follows.

As shown in the figure, the proposed method introduces a masking mechanism leveraging a KNN-derived approach to identify the most similar articles from the training set for each input article. It then ranks and extracts the most frequent MeSH terms from these similar articles as the candidate MeSH terms for each target article, which significantly reduces the complexity of the indexing problem. The extracted candidate MeSH terms are then embedded and fed into the downstream neural networks.

Moreover, the proposed neural network takes multiple textual components from different semantic aspects as inputs as well as the extracted candidate terms for each input article. These inputs are considered to be semantic probes and would be encoded under word embeddings and transformer encoders to generate further feature representations.

Additionally, the proposed neural network employs an attention mechanism to automatically assign different attentive weights to input probes and consequently attends to the most important semantic aspects of the input article. After the feature extraction at both term-level and document-level, the feature representations are further utilized to perform the following MeSH indexing prediction.

Finally, a linear multi-view classifier is adopted to take the extracted features from different semantic aspects to conduct the final MeSH classification. For each candidate term, the model is able to predict a probability score. In the training phase, the binary cross-entropy loss is utilized with a gradient-based method to optimize the model parameters. A more detailed description of the proposed method is provided in the following subsections.

### MeSH masking

COVID-19 semantic indexing is regarded as an extreme multi-label classification problem, which requires assigning appropriate labels from more than twenty thousand MeSH terms for each input article. How to reduce the high classification dimension is essential to the overall system performance. To tackle this problem, we employ a KNN algorithm to generate a refined subset of candidate terms for each input article. Technically, this generation procedure is considered to be MeSH Masking. The main ideas accounting for taking a small subset of candidate terms instead of the entire MeSH vocabulary are as follows: (i) Since each article merely carries around 13 MeSH annotations, there are far more negative terms than positive ones. The down-sampling of the negative samples is applicable by taking a recommended small subset of terms as candidates, in order that the classifier only needs to concentrate on predicting the most suitable terms from a plausible subset; (ii) During the training phase, a small subset of candidate terms is able to narrow down the prediction complexity as the neural network does not need to predict for the entire term vocabulary, which efficiently saves the model storage and calculation costs.

For each article, titles and abstracts are first split into a sequence of tokens, a word embedding matrix $E_{e} \in {\mathbb{R}}^{{|V_{e} | \times d_{e} }}$ is then utilized to convert all the tokens into low-dimensional dense vectors, where |*V*_*e*_| is the vocabulary size and *d*_*e*_ is the embedding size. In this regard, each input article can be represented by the sequence of word embeddings in accordance with its tokenized result, which can be consequently denoted as:

1 $${\varvec{D}} = [{\varvec{w}}_{1} , {\varvec{w}}_{2} , \ldots , {\varvec{w}}_{L} ] \in {\mathbb{R}}^{{L \times d_{e} }}$$

where ***D*** is viewed as a sequence of vectors that represents the input article. *L* is the sequence length and ***w***_*i*_ is the embedding vector for the word at position *i*. We further apply the KNN-driven strategy to choose the most similar articles from the training dataset for each input article. To this end, each article is represented by the Term Frequency-Inverse Document Frequency (TFIDF) weighted word embeddings:

2 $${\varvec{d}} = \frac{{\mathop {\mathbf{\sum }}\nolimits_{i = 1}^{L} tfidf_{i} \cdot {\varvec{w}}_{i} }}{{\mathop {\mathbf{\sum }}\nolimits_{i = 1}^{L} tfidf_{i} }} \in {\mathbb{R}}^{{d_{e} }}$$

Cosine similarity is adopted to find the most similar articles from the training set for each input article:

3 $${\text{Similarity}}(i, j) = \frac{{{\varvec{d}}_{i}^{T} {\varvec{d}}_{j} }}{{\left\| {{\varvec{d}}_{i} } \right\| \cdot \left\| {{\varvec{d}}_{j} } \right\|}}$$

After finding *K* nearest neighbors for each article, all MeSH terms in these neighbors are collected and ranked according to their frequency. In this way, top *M* MeSH terms are finally reserved as the candidate terms for each input article.

### Probe encoding

Regarding the abundance of meaningful representations from different semantic aspects, we propose to take advantage of multiple context-aware inputs of each article as semantic probes to extract potential biomedical clues for MeSH recommendations. Specifically, we mainly exploit four different semantic probes: Context Probe, Candidate Term Probe, Journal Probe, and Dynamic Topic Probe. We argue that each probe is able to carry certain semantic information of biomedical knowledge and fertilize the meaningful expression for each input article. The details of the above-mentioned semantic probes are introduced as follows:

#### Context probe

For each input article, its word sequence is considered to be the context probe, which conveys narrative textual information and offers implicit cues for determining MeSH recommendations. However, despite the meaningful representation of word embeddings, the word vectors are still less informative for text representation due to the lack of contextual comprehension. In this regard, a transformer encoder is adopted to read and encode the context probe as shown at the bottom of Fig. 3, which has shown promising results in many Natural Language Processing (NLP) areas [38–40]. This encoder makes use of both explicit and implicit textual correlations between the adjacent words. Specifically, each word in the context probe is represented by its hidden state generated from the encoder:

4 $${\varvec{t}}_{i} = {\text{Transformer}}(\theta ; w_{i} ) \in {\mathbb{R}}^{{d_{t} }}$$

where *θ* represents the parameters of the encoder, *d*_*t*_ stands for the hidden size, and ***t***_*i*_ is the encoded hidden state of the *i*-th word. The entire context probe is then represented accordingly by the sequence of the encoded hidden states, which is denoted as follows:

5 $${\varvec{T}} = [{\varvec{t}}_{1} ,{\varvec{t}}_{2} , \ldots , {\varvec{t}}_{L} ]^{T} \in {\mathbb{R}}^{{L \times d_{t} }}$$

where ${\text{T}} \in {\mathbb{R}}^{{L \times {\text{d}}_{{\text{t}}} }}$ is a *L*-by-*d*_*t*_ matrix concatenating all hidden states of words.

#### Candidate term probe

MeSH Masking procedure guarantees a handful subset with *M* most relevant terms for the recommendation, which are further taken as the candidate term probes for each input article. The refined small subset of candidate terms can notably mitigate the noise introduced by the extremely unbalanced negative term samples and provide a plausible semantic scope of topics to which the article pays attention. In practice, each term is taken as a single probe and is then converted through an embedding matrix ${\varvec{E}}_{f} \in {\mathbb{R}}^{{|V_{f} | \times d_{f} }}$, where |*V*_*f*_| is the vocabulary size and *d*_*f*_ is the embedding size. As word length usually differs in different term names, an RNN encoder is accordingly applied to acquire the name representation within a fixed length. In addition, in order to enhance the term representation, five kinds of statistical indicators are concatenated to the name representations, which are (a) a vector of length 2 indicating whether the candidate term occurs in the title and its frequency; (b) a vector of length 4 indicating whether the candidate term occurs in the first sentence, last sentence, and middle part of the abstract and its frequency; (c) a vector of length 2 indicating whether the candidate term can be recognized by MTI Online System [13, 22] and its score; (d) a vector of length 2 indicating whether the term is recognized by KNN and its score; (e) a scalar value indicating the global probability of term occurrence in the journal. The candidate term probes of the input article can be finally denoted as follows:

6 $${\varvec{H}} = [{\varvec{m}}_{1} , {\varvec{m}}_{2} , \ldots , {\varvec{m}}_{M} ]^{T} \in {\mathbb{R}}^{{M \times d_{f} }}$$

where ***m***_*i*_ is the probe representation of the *i*-th candidate term and *M* is the number of the recommended terms after the MeSH Masking stage.

#### Journal probe

In addition to Context Probe and Candidate Term Probe, Journal Probe is another informative semantic probe for MPANN. In the scientific area, articles are prone to be published in specific journals that are devoted to distinct research topics, such as chemicals, cancers, or coronavirus. This distinct information about journals is also important and instructive to provide essential cues for MeSH recommendations. To this end, each journal name that occurs in the corpus is taken as the journal probe. Specifically, each word in the journal probe is converted into a low-dimensional dense vector using the embedding matrix $E_{j} \in {\mathbb{R}}^{{|V_{j} | \times d_{j} }}$, where |*V*_*j*_| is the vocabulary size, and *d*_*j*_ is the embedding length. Since the word length is not identical among different journals, an RNN encoder is then leveraged to encode the word vectors to acquire the final hidden state ***c*** within a fixed length which is utilized to represent the journal probe.

#### Dynamic topic probe

Inspired by [25, 26], the dynamic topic probes are also introduced to the multi-probe attention neural network. Although MeSH Masking is able to sharply reduce the prediction space, some existing implicit yet general semantic aspects probably still exist beyond the scope of the current candidate term probes. For instance, an article dedicated to the new variant virus SARS-CoV-2 probably also discusses other general topics related to clinical treatments that might be missed in the candidate terms. Therefore, in order to capture this potential and meaningful topic information, a new kind of dynamic topic probe is proposed to represent additional informative topic aspects contained in the article. Compared with the candidate term probes which are explicitly related to some specific topics of the input article, the dynamic topic probes are more relevant to the general aspects of background knowledge beyond the candidate term probes. To this end, we employ the embedding matrix ${\varvec{E}}_{p} \in {\mathbb{R}}^{{|V_{p} | \times d_{p} }}$ to represent the *i*-th dynamic topic probe using a low-dimensional dense vector ***p***_*i*_, where |*V*_*p*_| is the vocabulary size and *d*_*p*_ is the size of the embedding vector. Accordingly, dynamic topic probes are inherent vectors of the model parameters, and each carries a certain aspect of general biomedical knowledge. Suppose there are *N* dynamic topic probes assigned to an input article, we can obtain the corresponding representation as an *N*-by-*d*_*p*_ matrix denoted as follows:

7 $${\varvec{P}} = [{\varvec{p}}_{1} , {\varvec{p}}_{2} , \ldots , {\varvec{p}}_{N} ]^{T} \in {\mathbb{R}}^{{N \times d_{p} }}$$

### Multi-probe attention

After encoding all the above-mentioned probes, we calculate the dot products among them to obtain the attended weight representations for different semantic aspects. The attentive feature representations at both the term-level and documental-level are primarily taken into consideration and further extracted for the downstream MeSH prediction. Specifically, we group these semantic probes into multiple pairs and calculate five different types of attention to obtain the attentive features. The calculation includes *Context-Term Attention*, *Journal-Term Attention*, *Journal-Context Attention*, *Journal-Topic Attention*, and *Context-Topic Attention*.

#### Feature representation at term level

For feature representation at the term level, we separately represent and extract the attentive features by calculating Context-Term Attention and Journal-Term Attention. For Context-Term Attention, given the encoded context probes ***T*** and candidate term probes ***H***, we first compute their attentive weight matrix ***G*** and then adopt a SoftMax function to get the normalized attention weights as follows:

8 $${\varvec{G}} = \left[ {{\varvec{Tm}}_{1} , {\varvec{Tm}}_{2} , \ldots ,{\varvec{Tm}}_{M} } \right]^{T} \in {\mathbb{R}}^{M \times L}$$

9 $${\varvec{\alpha}}_{i}^{G} = SoftMax\left( {{\varvec{Tm}}_{i} } \right) \in {\mathbb{R}}^{L}$$

10 $$SoftMax\left( {\varvec{G}} \right) = [{\varvec{\alpha}}_{1}^{G} ,{\varvec{\alpha}}_{2}^{G} , ..., {\varvec{\alpha}}_{M}^{G} ]^{T} \in {\mathbb{R}}^{M \times L}$$

where ${\varvec{\alpha}}_{i}^{G} \in [0, 1]^{L}$ is the *i-*th weight vector over the context probe ***T*** and $\sum_{k = 1}^{L} \alpha_{ik}^{G} = 1$. Technically, the higher the weight value, the more related the attention is paid to the probe. Each term-specific representation is then computed by the attentive weight vectors and textual probes:

11 $${\varvec{e}}_{i}^{G} = {[}{\varvec{\alpha}}_{i}^{G} ]^{{\text{T}}} {\text{T}} \in {\mathbb{R}}^{{{\text{d}}_{{\text{t}}} }}$$

where ${\varvec{e}}_{i}^{G}$ is *i-*th term-aware specific representation. The term-aware contextual feature ${\varvec{e}}^{G} \in {\mathbb{R}}^{{d_{t} }}$ is the mean value of the summation of $\sum_{{\text{i = 1}}}^{{\text{M}}} {\varvec{e}}_{i}^{G}$.

For Journal-Term Attention, we calculate and extract the term-aware feature in the same way as follows:

12 $${\varvec{\alpha}}^{J} = SoftMax\left( {{\varvec{Hc}}} \right) \in {\mathbb{R}}^{M}$$

13 $${\varvec{e}}^{J} = [{\varvec{\alpha}}^{J} ]^{T} {\varvec{H}} \in {\mathbb{R}}^{{d_{m} }}$$

where ${\varvec{\alpha}}^{J} \in [0, 1]^{M}$ is the attention weight over the term probe ***m***_*i*_ and ${\varvec{e}}^{J} \in {\mathbb{R}}^{{d_{m} }}$ is the feature representation. We concatenate the extracted feature vectors ***e***^*G*^ and ***e***^*J*^ into the vector ***r***^*T*^ as the feature representation for the term level.

#### Feature representation at documental level

Apart from the feature extraction at the term level, we also propose to extract the features from the document level. Particularly, we extract the attentive features through Context-Topic Attention, Journal-Context Attention, and Journal-Topic Attention, respectively. Given the encoded probes ***T*** and ***P***, we extract the topic-aware contextual feature by computing the Context-Topic Attention. The calculations are denoted as follows:

14 $${\varvec{U}} = [{\varvec{Tp}}_{1} , {\varvec{Tp}}_{2} , \ldots ,{\varvec{Tp}}_{N} ]^{T} \in {\mathbb{R}}^{N \times L}$$

15 $${\varvec{\alpha}}_{i}^{U} = SoftMax\left( {{\varvec{Tp}}_{i} } \right) \in {\mathbb{R}}^{L}$$

16 $${\varvec{e}}_{i}^{U} = [{\varvec{\alpha}}_{i}^{U} ]^{T} {\varvec{T}} \in {\mathbb{R}}^{{d_{t} }}$$

where ***U*** is the weight matrix, ${\varvec{\alpha}}_{i}^{U} \in [0, 1]^{L}$ is the weight vector over the context probes, and $\sum_{k = 1}^{L} \alpha_{ik}^{U} = 1$; ${\varvec{e}}_{i}^{U}$ is *i-*th topic specific representation. The topic-aware contextual feature ${\varvec{e}}^{U} \in {\mathbb{R}}^{{d_{t} }}$ is represented using the mean value of the summation of $\sum_{i = 1}^{Q} {\varvec{e}}_{i}^{U}$.

Similarly, features encoded by Journal-Topic Attention and Journal-Context Attention are extracted in the same way as follows:

17 $${\varvec{\alpha}}^{S} = SoftMax\left( {{\varvec{Pc}}} \right) \in {\mathbb{R}}^{N}$$

18 $$e^{S} = [{\varvec{\alpha}}^{S} ]^{T} {\varvec{P}} \in {\mathbb{R}}^{{d_{p} }}$$

19 $${\varvec{\alpha}}^{Q} = SoftMax\left( {{\varvec{Tc}}} \right) \in {\mathbb{R}}^{N}$$

20 $${\varvec{e}}^{Q} = [{\varvec{\alpha}}^{Q} ]^{T} {\varvec{T}} \in {\mathbb{R}}^{{d_{t} }}$$

where ${\varvec{\alpha}}^{S} \in [0, 1]^{N}$ and ${\varvec{\alpha}}^{S} \in [0, 1]^{N}$ are the normalized weight vectors over the dynamic topic probes and context probes, respectively; ${\varvec{e}}^{S} \in {\mathbb{R}}^{{d_{p} }}$ and ${\varvec{e}}^{Q} \in {\mathbb{R}}^{{d_{t} }}$ are the respective feature representations. The extracted feature vectors ***e***^*U*^*, ****e***^*S*^ and ***e***^*J*^ are concatenated into the vector ***r***^*D*^ which is considered as the feature representation for the document level.

### Multi-view classification

Benefiting from the attention mechanism, the feature representations at both term level and document level are finally extracted. To compute the confidence of MeSH recommendation, the feature representations ***r***^*T*^ and ***r***^*D*^ are further concatenated to form the final feature vector ***v*** and are fed into the linear projection layer with a *Sigmoid* activation function. The final output ${\varvec{o}} \in {\mathbb{R}}^{M}$ is used to calculate the probability score for each corresponding MeSH term:

21 $${\varvec{o}} = \sigma ({\varvec{Wr}} + {\varvec{b}})$$

where ${\varvec{W}} \in {\mathbb{R}}^{{M \times d_{v} }}$ is the linear transformation matrix, ${\varvec{b}} \in {\mathbb{R}}^{M}$ is the bias, and *σ* is the *Sigmoid* activation function. The value *M* equals the number of the candidate MeSH terms for the classification and each output can be interpreted as the confidence score of the corresponding recommendation.

To learn the parameters of the network, the binary cross-entropy loss function is used via the calculation of the predicted terms and the gold MeSH annotations in the training set:

22 $${\mathcal{L}}_{j} = - (y_{j} \log (\hat{y}_{j} ) + (1 - y_{j} )\log (1 - \hat{y}_{j} ))$$

where $y_{j} \in [0, 1]$ is the ground-truth label of the *j*-th MeSH term; *y*_*j*_ = 0 means the *j*-th MeSH term is not annotated to the article by human indexers, while *y*_*j*_ = 1 means the *j*-th MeSH term is annotated. We can calculate the total loss by summing them up:

23 $${\mathcal{L}} = \mathop \sum \limits_{j = 1}^{M} {\mathcal{L}}_{j}$$

The entire framework of MPANN is trained end-to-end by a gradient-based optimization algorithm to minimize the loss of ${\mathcal{L}}$.

## Results

In this section, we first introduce the evaluation metrics and the experimental settings for COVID-19 semantic indexing; we then systematically evaluate MPANN on the CovSI corpus and compare it with the state-of-the-art systems. Furthermore, to verify the effectiveness and generalizability of MPANN, we perform additional experiments on the BioASQ Task9a dataset and compare it with highly relevant systems. Finally, we conduct the error analysis at the end of this section.

### Evaluation metrics

Generally, there is no such unified evaluation standard for COVID-19 semantic indexing, which is essentially a multi-label classification problem. In this research, following the previous works [25–29], we adopted the evaluation metrics proposed by BioASQ [9] to evaluate our proposed method.

Let *K* denote the size of all MeSH labels (i.e. MeSH terms), and *N* denotes the number of the input instances (i.e. biomedical articles). Let *y*_*i*_ and $\hat{y}_{i} \in \{ 0, 1\}^{K}$ be the true and predicted labels for instance *i*, respectively. We mainly adopted three different metrics based on F-measure at different levels to evaluate the performance of our models.

#### Example-based F-measure (EBF):

EBF is utilized to evaluate the system performance at the instance level. EBF can be computed by the harmonic mean of example-based precision (EBP) and example-based recall (EBR) as follows:

24 $${\text{EBF}} = \frac{1}{N}\mathop \sum \limits_{i = 1}^{N} {\text{EBF}}_{i}$$

where

25 $${\text{EBF}}_{i} = \frac{{2 \cdot {\text{EBP}}_{i} \cdot {\text{EBR}}_{i} }}{{{\text{EBP}}_{i} + {\text{EBR}}_{i} }}$$

where

26 $${\text{EBP}}_{i} = \frac{{\mathop \sum \nolimits_{k = 1}^{K} y_{i}^{k} \cdot \hat{y}_{i}^{k} }}{{\mathop \sum \nolimits_{k = 1}^{K} \hat{y}_{i}^{k} }}\quad {\text{EBR}}_{i} = \frac{{\mathop \sum \nolimits_{k = 1}^{K} y_{i}^{k} \cdot \hat{y}_{i}^{k} }}{{\mathop \sum \nolimits_{k = 1}^{K} y_{i}^{k} }}$$

Note that EBP and EBR are calculated by summing EBP_*i*_ and EBR_*i*_ over all instances, respectively.

#### Macro F-measure (MaF)

MaF is utilized to evaluate the system performance at the macro level of labels. In MaF, all the labels are treated equally regardless of their distribution. MaF can be computed by the harmonic mean of macro-average precision (MaP) and macro-average recall (MaR) as follows:

27 $${\text{MaF}} = \frac{{2 \cdot {\text{MaP}} \cdot {\text{MaR}}}}{{{\text{MaP}} + {\text{MaR}}}}$$

The macro-average precision and recall are obtained by first computing the precision and recall for each label (i.e. Mesh term) separately, and then averaging them over all labels as follows:

28 $${\text{MaP}} = \frac{1}{K}\mathop \sum \limits_{k = 1}^{K} P^{k} \quad {\text{MaR}} = \frac{1}{K}\mathop \sum \limits_{k = 1}^{K} R^{k}$$

where

$$P^{k} = \frac{{\mathop \sum \nolimits_{i = 1}^{N} y_{i}^{k} \cdot \hat{y}_{i}^{k} }}{{\mathop \sum \nolimits_{i = 1}^{N} \hat{y}_{i}^{k} }}\quad R^{k} = \frac{{\mathop \sum \nolimits_{i = 1}^{N} y_{i}^{k} \cdot \hat{y}_{i}^{k} }}{{\mathop \sum \nolimits_{i = 1}^{N} y_{i}^{k} }}$$

#### Micro F-measure (MiF):

MiF is utilized to evaluate the system performance at the micro level of labels. In MiF, the distribution of each label is taken into consideration, and the labels with larger numbers are more influential to the final results during the calculation. MiF can be computed by the harmonic mean of micro-average precision (MiP) and micro-average recall (MiR) as follows:

29 $${\text{MiF}} = \frac{{2 \cdot {\text{MiP}} \cdot {\text{MiR}}}}{{{\text{MiP}} + {\text{MiR}}}}$$

where

$${\text{MiP}} = \frac{{\mathop \sum \nolimits_{k = 1}^{K} \mathop \sum \nolimits_{i = 1}^{N} y_{i}^{k} \cdot \hat{y}_{i}^{k} }}{{\mathop \sum \nolimits_{k = 1}^{K} \mathop \sum \nolimits_{i = 1}^{N} \hat{y}_{i}^{k} }}\quad {\text{MiR}} = \frac{{\mathop \sum \nolimits_{k = 1}^{K} \mathop \sum \nolimits_{i = 1}^{N} y_{i}^{k} \cdot \hat{y}_{i}^{k} }}{{\mathop \sum \nolimits_{k = 1}^{K} \mathop \sum \nolimits_{i = 1}^{N} y_{i}^{k} }}$$

As is suggested by BioASQ [9], among all the evaluation metrics, MiF is the crucial evaluation criterion for determining the overall quality of the involved system.

### Experimental settings

Following previous works [25, 26], in the preprocessing stage, all non-alphanumeric characters, stop words, low-frequency words occurring less than five times are removed, and all tokens are converted into lowercase. In case article texts partially exceed the length limitations of the transformer encoder, the head and tail parts of the overlong texts are reserved as the final input texts instead of the original ones. For initialization, the word vectors provided by the BioASQ community are utilized to initialize the word embeddings ***E***_*e*_, other parameters in the model are randomly initialized. The AdamW optimizer [41] is used to minimize the training loss and the settings of the hyper-parameter are listed in Table 3.

**Table 3:** The settings of the hyper-parameters

Parameter	Value
Batch size	10
Word embedding size *d*_*e*_	200
Sequence length *L*	512
Transformer hidden size *d*_*t*_	200
Candidate term size *M*	400
Term embedding size *d*_*f*_	200
Journal embedding size *d*_*j*_	200
Dynamic topic probe size *N*	30
Dynamic probe embedding size *d*_*p*_	200
Linear layer size *d*_*v*_	200
Dropout rate	0.3
Learning rate	0.00001

The model of MPANN is pre-trained with 2 million latest biomedical articles, which are extracted from MEDLINE with the goal of learning general biomedical knowledge. It is then fine-tuned on the training and development datasets of CovSI to learn the domain-specific knowledge of COVID-19. Once the parameters and hyper-parameters are well-tuned, MPANN is used to perform the topic prediction for the CovSI test set. The entire training process of MPANN takes approximately 8 days and requires 4 NVIDIA 2080 graphic cards.

### Comparison with related systems

In the following section, a comprehensive comparison among four relevant state-of-the-art systems [26, 28–30] and MPANN is performed. Since the compared systems are not originally designed for the topic of COVID-19, we transferred and re-trained these systems for the COVID-19 domain. Note that the default settings of these systems reported in previous research are followed during the comparison. Additionally, all models are trained in the same way as MPANN, which means these models are first pre-trained with an external large-scale data of 2 million MEDLINE articles, and then fine-tuned on the basis of the CovSI training and development sets. After the training phase, these models are exploited to perform the prediction on the CovSI test set. The overall performance of the above-mentioned systems is summarized in Table 4 and the highest scores of F-measures are bolded.

**Table 4:** The comparison of different systems on the CovSI test set

Model	EBP (%)	EBR (%)	EBF (%)	MaP (%)	MaR (%)	MaF (%)	MiP (%)	MiR (%)	MiF (%)
MPANN	87.41	63.52	**71.20**	97.03	50.44	55.02	88.62	62.78	**73.49**
BioTrans [30]	87.02	62.74	70.47	97.17	47.63	52.23	87.99	61.92	72.68
AttentionMeSH [29]	81.18	54.52	63.08	88.51	51.15	54.36	81.57	53.48	64.60
FullMeSH [28]	88.40	51.92	63.29	95.11	57.56	60.47	88.44	51.92	65.43
MeSHProbeNet-P [26]	82.81	54.36	65.64	95.64	57.66	**61.29**	83.33	57.14	67.79

As shown in the table, the CNN-based neural attention model FullMeSH and the RNN-based neural attention model AttentionMeSH obtain comparable performance, which results in the MiF of 65.43% and 64.60%, respectively. This is likely due to the similar representation capabilities of both CNN and RNN, which are able to capture effective semantic information from contextual texts. However, compared to FullMeSH, AttentionMeSH has higher recall but much lower precision, resulting in a relatively lower F-score. MeSHProbeNet-P consistently outperforms FullMeSH and AttentionMeSH in terms of all F-measures with the MiF as high as 67.79%, while its precision is slightly lower than FullMeSH, which suggests MeSHProbeNet-P pays more attention to the coverage of MeSH terms. This is probably because MeSHProbeNet-P leverages the entire MeSH vocabulary to train and predict, which increases the chance of learning more comprehensive correlations between terms and contextual words. However, since MeSH terms carry a huge vocabulary, using that large set of MeSH terms to train is rather time-consuming. BioTrans shows superior performance in all state-of-the-art systems. Due to the powerful representation ability of the pre-trained structure, BioTrans achieves an MiF performane as high as 72.68%. Since MPANN is able to capture the correlations between the MeSH terms and their contextual inputs, MPANN rivals the other systems and achieves the highest MiF and EBF with scores of 73.49% and 71.20%, respectively. However, compared with FullMeSH and MeSHProbeNet-P, MPANN acquires a relatively lower MaF performance. This implies that MPANN may have the tendency to pay more attention to the imbalanced term distribution and predict the head terms aggressively and the tail terms conservatively. It is worth mentioning that, compared with BioTrans which is also the pre-trained model for COVID-19, MPANN consistently outperforms all F-scores improving by 0.73% in EBF, by 2.79% in MaF, and by 0.81% in MiF, respectively. This indicates that the multi-probe attention mechanism is able to provide more robust COVID-19 specific feature representations which can benefit the ultimate semantic indexing performance.

### Feature ablation performance

To investigate the importance of the contributions of the proposed semantic probes, we perform the ablation studies of MPANN as follows. One of the core claims is that the representations of the probes are able to provide comprehensive biomedical background information, which is crucial for the topic of COVID-19 semantic indexing. To verify the assumptions, we compare the default version of MPANN (i.e. MPANN-*Default*) with its variants without the probe attentions described in the *Method* section, trying to reveal the different impacts of the semantic probes. It is worth recognizing that the large external pre-training step should be emphasized, which significantly improves the overall system performance. In comparison, the naive version of MPANN (MPANN-*Naive*) is also performed which is merely trained based on the small scale of the CovSI corpus without any external training data. All the other hyper-parameters of the model are kept identical during the comparison. Table 5 exhibits the details of the ablated experimental comparison, in which the highest scores are highlighted in boldface.

**Table 5:** The ablation experiments of MPANN

Model name		EBP (%)	EBR (%)	EBF (%)	MaP (%)	MaR (%)	MaF (%)	MiP (%)	MiR (%)	MiF (%)
MPANN	MPANN-*Default*	87.41	**63.52**	**71.20**	97.03	50.44	**55.02**	88.62	**62.78**	**73.49**
	- Context-Term Attention	94.86	52.90	64.25	98.70	41.51	46.28	93.50	52.97	67.64
	- Journal-Term Attention	90.66	60.78	70.28	98.36	47.63	52.58	91.55	59.71	72.28
	- Journal-Context Attention	86.59	59.85	68.29	96.51	47.78	52.29	88.39	59.32	70.99
	- Journal-Topic Attention	87.61	62.29	70.36	97.06	49.40	54.01	88.88	61.60	72.77
	- Context-Topic Attention	84.91	62.47	69.64	96.02	**50.40**	54.87	86.57	62.05	72.29
	MPANN-*Naive*	**97.17**	41.89	54.76	**98.89**	36.06	40.78	**98.02**	42.81	59.60

In general, as can be observed from the table, the default version of MPANN consistently outperforms its ablations without attention modeling. Furthermore, it is observed that without the Context-Term Attention, the final performance of MiF drops drastically to the score of 67.64%. This suggests that modeling the correlations between the contextual information and the candidate terms is crucial for COVID-19 semantic indexing. Likewise, the models without Journal-Term Attention, Journal-Topic Attention, or Context-Topic Attention perform comparably with slight decline in the scores of MiF. This implies that all the probes of journals, candidate terms, dynamic topics, and contexts carry specific biomedical informative aspects, allowing the models to effectively couple the correlations among them, which benefit the overall performance for COVID-19 semantic indexing. Since journal probes can carry specific topics related to biomedical background knowledge, modeling the correlations between the journal probes and context probes is also important for MPANN, the absence of which leads to an overall decrease of 2.5 points in the score of MiF. For a fair comparison, we also compare the naive version MPANN-*Naive* which is only trained on the CovSI dataset without any external data. In Table 5, we find that MPANN-*Naive* performs the worst, indicating its limited learning capability. Compared to other models, although MPANN-*Naive* acquires higher precision, its recalls exhibit much worse results. This is likely because of the extremely sparse term distribution which makes it difficult to learn essential representations when only using a limited amount of the training data. In contrast, utilizing a large number of external data in model pre-training, MPANN-*Default* can guarantee abundant priori biomedical knowledge which lays the foundation for the learning capability. Once adapted to the COVID-19 domain, the pre-trained knowledge can help the MPANN model to more effectively learn the specific knowledge related to COVID-19.

To investigate the impacts of the hyper-parameters, we evaluate the effects with different settings. The hyper-parameters *M* and *N* are primarily taken into consideration, which stands for the number of candidate term probes and dynamic topic probes, respectively.

**Fig. 4:**
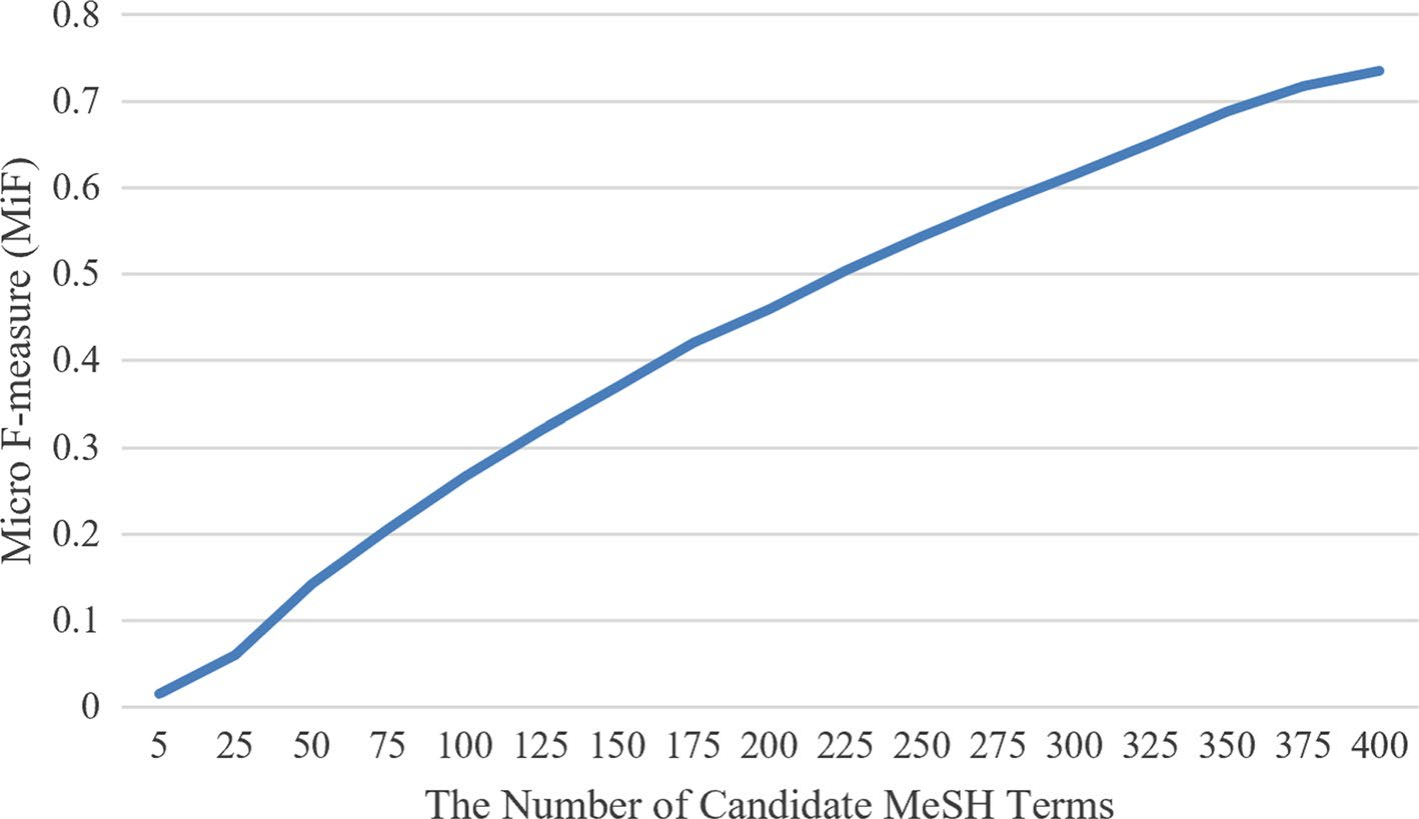
The performance of MiF with different settings of hyper-parameter *M*

Figure 4 depicts the effect of the hyper-parameter *M* with different settings on the CovSI test dataset. Note that when *M* is tuning, all the other hyper-parameters remain the same as described in the section of *Experimental Settings*. From the figure, it can be observed that the performance rises stably along with the increase of the hyperparameter *M* and reaches the best performance with the value of 400 at last. This indicates that by enlarging the number of candidate MeSH terms with a relatively larger *M*, the model can increase the coverage of the true terms, resulting in a significant improvement in the measurement of recall. However, an excessive increase of *M* requires more computing resources and introduces more unexpected noise leading to increased training difficulty. To this end, we set the maximum value to 400 for the hyper-parameter *M* in our experiments.

**Fig. 5:**
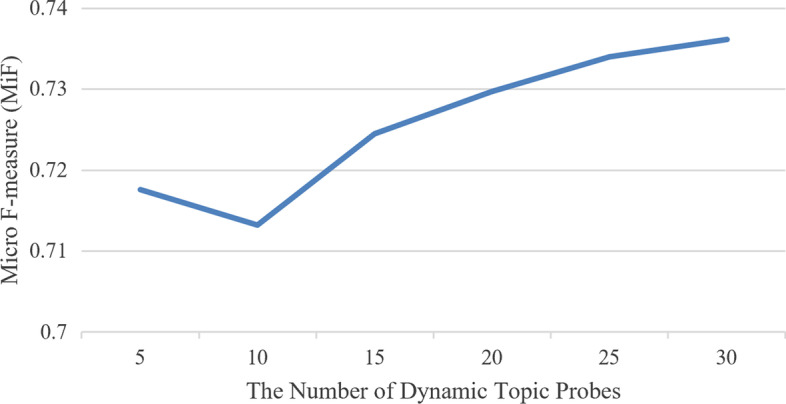
The performance of MiF with different settings of hyper-parameter *N*

Figure 5 illustrates the effect of the hyper-parameter *N* related to the different settings of dynamic topic probes on the CovSI test dataset. Note that MPANN models with 5, 15 20, 25, and 30 dynamic topic probes are included in the comparison. When the hyper-parameter *N* is changing, all the other hyper-parameters stay the same as described in the section of *Experimental Settings*. In Fig. 5, it is observed that with the increase of *N*, there is a slight decline of MiF at first, and then the performance rises consistently as *N* increases further until it reaches around 30. This is probably because increasing the number of the dynamic topic probes can robustly reflect some general topic aspects, and MPANN can effectively grasp such kind of semantic feature representations. However, it seems that overmuch information on dynamic topics cannot provide more meaningful clues for COVID-19 semantic indexing, which is unable to further improve the overall performance.

### Robustness of MPANN: a study based on the BioASQ dataset

Since MPANN is developed and tested on the relatively small and domain-specific CovSI corpus, the reported success might not provide sufficient evidence for the robustness of the proposed multiprobe attention model. To corroborate the claim of the robustness of the multiprobe attention model, we conduct an independent study of MPANN on the BioASQ Task9a dataset [9]. It is worth noting that the BioASQ dataset is widely accepted for system evaluation in topics of semantic indexing due to the large-scale data size and the comprehensive topic coverage. In particular, BioASQ Task9a provides an extremely large-scale dataset consisting of 15,559,157 training articles and 90,724 test articles, respectively. Each article in the dataset carries around 12 semantic topics on average, and the test set is further divided into 15 separate batches for the online competition.

Compared with the CovSI corpus, as BioASQ Task9a provides a significant scale-up in the topic coverage with a much larger data size, the discrepancies are mainly underlined by the fact that more than 10 thousand types of MeSH semantic topics in BioASQ are never attested in the CovSI corpus, which inevitably aggravates the difficulties of the model adaptation. To ensure that the result can be comparable, we thus re-trained the model on the new dataset of BioASQ Task9a. Since the goal is to support the robustness of the proposed model, we simply adopt the widely used pre-trained model of BioBERT [40] for the initialization. It is worth noticing that, during the training phase, terms and journals will share the same word vocabulary with the pre-trained model. Table 6 compares MPANN with the state-of-the-art systems that participated in the BioASQ Task9a [9]. Since a few teams made multiple submissions, the best-performed ones are listed for comparison. All the experimental results reported in the table are averaged on the 15 different test batches. As shown in the table, the model of *deepmesh_dmiip_fdu* achieves the best performance in all F-measures, resulting in the highest EBF of 68.87%, MaF of 58.69, and MiF of 69.32%, respectively. Moreover, it is also observed that most performance scores of MiF are higher than 60%, while the MiF measures of *bert_dna* and *iria-1* are relatively lower. As the current study is to establish the robustness and generalizability of MPANN, it is thus reasonable to expect a robust model trained for other tasks to achieve comparable performance. In Table 6, compared with the state-of-the-art systems, MPANN reaches competitive precision scores close to the top system and obtains modest recall scores slightly lower than the top submissions. Note that the proposed MPANN model is designed for a new specific domain with built-in robustness that consists of a multitude of heterogeneous issues but shares a number of the same points of attention related to one single topic, i.e., COVID-19. Therefore, the methodology is not optimized for other broader fields with a significant range of diverse points of attention, such as BioASQ. However, although the MPANN model does not perform as high as the best systems, it still reaches a comparable performance with a promising score of 64.59% in the MiF measure, indicating the effectiveness and generalizability. This performance is, in fact, consistent with its original design for identifying semantic topics from a specific emerging field. In addition, the detailed performance on all batches of the test data, shown in Table 7, reassures that MPANN is well balanced and not overfitted to favor any particular field.

**Table 6:** The comparison of the state-of-the-art systems on the BioASQ test set

System	EBP(%)	EBR(%)	EBF(%)	MaP(%)	MaR(%)	MaF(%)	MiP(%)	MiR(%)	MiF(%)
deepmesh_dmiip_fdu	72.51	**68.69**	**68.87**	70.10	59.34	**58.69**	72.02	**66.86**	**69.32**
NLM System 3	71.28	67.87	67.74	69.22	54.67	54.53	71.01	65.94	68.37
attention_dmiip_fdu	68.40	65.65	65.40	65.53	55.84	55.06	67.95	63.87	65.84
MTI First Line Index	69.39	63.58	64.50	65.43	57.33	55.38	68.21	61.52	64.69
Default MTI	64.54	67.28	64.02	61.17	**60.55**	56.54	63.76	65.11	64.42
NLM CNN	68.03	62.11	62.86	63.02	45.81	46.10	67.30	60.75	63.85
pi_dna_3	65.73	62.45	62.14	55.50	50.37	48.45	65.01	60.75	62.80
bert_dna	61.31	55.15	56.02	48.86	38.52	37.05	60.57	53.90	57.03
iria-1	41.70	55.25	46.36	38.92	39.16	35.14	42.11	53.89	47.28
MPANN	72.13	59.73	63.70	68.13	52.62	53.20	72.02	58.56	64.59

**Table 7:** The detailed performance of MPANN on different batches of the BioASQ test set

Batch	#Articles	EBP(%)	EBR(%)	EBF(%)	MaP(%)	MaR(%)	MaF(%)	MiP(%)	MiR(%)	MiF(%)
Test batch 1, week 1	7,967	73.36	60.56	64.78	68.88	53.52	54.15	73.33	59.41	65.64
Test batch 1, week 2	10,053	71.32	58.97	62.76	67.72	53.04	53.65	71.06	57.58	63.61
Test batch 1, week 3	4,870	72.04	60.31	64.05	67.49	52.43	52.86	71.92	59.06	64.86
Test batch 1, week 4	5,758	71.81	58.79	62.94	68.15	52.29	53.08	71.87	57.52	63.90
Test batch 1, week 5	5,770	70.81	60.34	63.48	66.77	52.13	52.25	70.43	59.41	64.45
Test batch 2, week 1	6,376	72.11	58.15	62.60	68.40	51.11	52.02	72.04	57.00	63.64
Test batch 2, week 2	9,101	70.93	58.29	62.31	67.74	53.08	53.63	70.99	57.48	63.52
Test batch 2, week 3	7,013	71.88	58.30	62.81	68.05	52.74	53.41	71.81	57.25	63.71
Test batch 2, week 4	6,070	73.35	59.64	64.17	69.31	53.35	53.93	73.04	58.51	64.98
Test batch 2, week 5	6,151	73.14	61.17	64.94	68.70	53.02	53.76	73.16	59.84	65.83
Test batch 3, week 1	5,890	73.31	60.09	64.59	68.69	51.59	52.46	73.31	59.20	65.51
Test batch 3, week 2	10,818	72.61	59.10	63.55	68.27	53.05	53.97	72.38	57.84	64.30
Test batch 3, week 3	4,022	71.43	60.79	64.07	68.46	50.86	51.38	71.61	59.50	65.00
Test batch 3, week 4	5,373	72.89	60.93	64.80	68.25	52.82	53.35	72.62	59.45	65.38
Test batch 3, week 5	5,325	70.93	60.53	63.63	67.08	54.26	54.17	70.70	59.36	64.54

### Error analysis

To provide insights for future work of COVID-19 semantic indexing, we closely analyzed the prediction errors from the article perspective and grouped the main reasons as follows:

(i) ***Imprecise candidate term selection***: This kind of prediction error happens in around 36% of the wrongly predicted articles in the CovSI corpus. Although MeSH Masking is able to considerably narrow down the large MeSH vocabulary into a small subset for the downstream prediction, it inevitably misses some critical terms on account of the limitation of the KNN-derived recommendation. Actually, after the MeSH Masking phase, the coverage of candidate terms for each article is only around 92% on average, which indicates the remaining 8% of the ground truth answers of an article will never be observed by MPANN. For instance, in the article PMID:33,213,707, the KNN-based approach provides the candidate terms of ‘*Pandemics’*, ‘*COVID-19*’, and ‘*SARS-Cov-2*’, which could be correctly predicted by MPANN; however, the low-frequent term of ‘Denture, Overlay’ that is not relevant to COVID-19 cannot be recognized as it is missed by the stage of MeSH Masking.
(ii) ***MeSH Masking Noise***: In spite of the fact that MeSH Masking tries the best to provide a small subset of reliable candidate terms, it still introduces ranking noises to the downstream pipelines, resulting in false labels with much higher confidence while true labels on the opposite. This kind of error dominates the most majority of the prediction errors and happens in almost 82% of the wrongly predicted articles. For instance, the typical term of ‘*Clinical Competence*’ cannot be predicted in the article of PMID:33,222,986 as the term is provided with relatively lower confidence by MeSH Masking.
(iii) ***Insufficient textual contents***: Since our experiments only take the titles and abstracts of articles into consideration when exploring the COVID-19 semantic indexing problems, these limited textual inputs may miss some critical clues that occur in the body text. Taking the article of PMID:32,951,723 for example, its topic mainly focuses on the combined therapy of COVID-19, however, none of the medicine-related MeSH terms such as ‘*Indoles’*, ‘*Lopinavir’*, ‘*Moxifloxacin*’, ‘*Methylprednisolone*’, and ‘*Anti-Bacterial Agents*’ occurs in the title or abstract. On the contrary, all of these concepts occur in the body text of the article. As there is no such evidence carried by its title and abstract, the MPANN model cannot correctly predict these medicine-related terms. This kind of error takes place in around 18% of the wrongly predicted articles in the corpus.
(iv) ***Complexity of language expression***: In some cases, if multiple similar MeSH terms are simultaneously provided as candidates, it would be difficult for MPANN to distinguish when lacking explicit evidence in the input contexts. For instance, in the article *PMID:33,222,986,* our MPANN cannot precisely recognize the true terms of *‘Orthopedic Procedures’ and ‘Orthopedic Surgeons’,* while it identifies another term of *‘Orthopedic’*, which is the hypernym term of both *‘Orthopedic Procedures’ and ‘Orthopedic Surgeons’*. This kind of error happens in around 47% of the wrongly predicted articles in the corpus.
(v) ***Inconsistent annotation***: In our experiments, it seems that some supposedly false-positive MeSH topic terms identified by MPANN may be actually correct and should be annotated in the corpus. For instance, in the article PMID:32,539,372, the terms ‘*Betacoronavirus’*, ‘*Coronavirus 3C Proteases*’, and ‘*SARS-CoV-2*’ are indeed annotated, while the typical term ‘*COVID-19*’ is not annotated but identified by MPANN. These kinds of errors, due to the inconsistent annotations, are around 21% of the wrongly predicted articles in the corpus. It is well known that the MEDLINE curation with MeSH headings inevitably contains some human errors. The fact that our system can identify the mislabeled terms underlines the robustness of the proposed approach. Meanwhile, these findings may also provide some feedback for further refinement of MeSH annotations in the future.

## Conclusions and future work

This research provided a new benchmark dataset and a novel multi-probe attention approach for COVID-19 semantic indexing. To exploit the efficiency of our proposed model, we first construct the CovSI corpus focusing on the COVID-19 topic, we then leverage the proposed model to address the COVID-19 semantic indexing problem. In the proposed approach MPANN, we use a KNN-derived MeSH masking mechanism to generate a handful of candidate MeSH terms for each input article; we then encode and feed the candidate terms as well as other textual information as probes into the downstream attention-based neural network. After extracting the semantic feature representations at both term level and document level, our MPANN model adopts a linear multi-view classifier to conduct the final MeSH term prediction. The experimental results suggest the effectiveness of our proposed approach.

Our research on deep learning exhibits promising results for the COVID-19 semantic indexing research on biomedical literature. In future work, we plan to develop more advanced deep learning algorithms with richer representation capabilities and extend the corpus to other domains and languages for better generalization.

## Data Availability

The resources of PMC and MEDLINE can be found at https://pubmed.ncbi.nlm.nih.gov and https://www.ncbi.nlm.nih.gov/pmc/tools/ftp. The CORD-19 dataset is located at https://allenai.org/data/cord-19. The BioASQ datasets can be found at http://www.bioasq.org. The CovSI dataset and codes are available at https://github.com/JHnlp/MPANN/.

## Abbreviations

MeSH: Medical subject headings
CovSI: COVID-19 semantic indexing
CORD-19: COVID-19 open research dataset
MPANN: Multi-probe attention neural network
KNN: K-nearest neighbor
NLM: National library of medicine
PMC: PubMed central
LTR: Learning to rank
MTI: Medical text indexing
D2V: Document to vector
TFIDF: Term frequency with inverse document frequency
CNN: Convolution neural network
SVM: Support vector machine
RNN: Recurrent neural network
NLP: Natural language processing
EBF: Example-based F-measure
EBP: Example-based precision
EBR: Example-based recall
MaF: Macro F-measure
MaP: Macro-average precision
MaR: Macro-average recall
MiF: Micro F-measure
MiP: Micro-average precision
MiR: Micro-average recall
